# Quantitative biometric phenotype analysis in mouse lenses

**Published:** 2010-06-08

**Authors:** Matthew A. Reilly, Usha P. Andley

**Affiliations:** 1 Department of Veterans Affairs Medical Center, St. Louis, MO; 2 Energy, Environmental, and Chemical Engineering, Washington University in St. Louis, St. Louis, MO; 3 Ophthalmology and Visual Sciences, Washington University in St. Louis, St. Louis, MO

## Abstract

The disrupted morphology of lenses in mouse models for cataracts precludes accurate in vitro assessment of lens growth by weight. To overcome this limitation, we developed morphometric methods to assess defects in eye lens growth and shape in mice expressing the αA-crystallin R49C (αA-R49C) mutation. Our morphometric methods determine quantitative shape and dry weight of the whole lens from histological sections of the lens. This method was then used to quantitatively compare the biometric growth patterns of lenses of different genotypes of mice from birth to 12 months. The wild type dry lens weights determined using the morphometric method were comparable to previously reported weights. Next we applied the method to assessing the lenses of αA-R49C knock-in mice, which exhibit decreased αA-crystallin protein solubility, resulting in a variety of growth abnormalities including early cataract formation, decreased eye and lens size, failure to form the equatorial bow region, and continued lens cell death, sometimes resulting in the entire loss of the lens and eye. Our morphometric methods reproducibly quantified these defects by combining histology, microscopy, and image analysis. The volume measurement accurately represented the total growth of the lens, whereas the geometric shape of the lens more accurately quantified the differences between the growth of the mutant and wild-type lenses. These methods are robust tools for measuring dry lens weight and quantitatively comparing the growth of small lenses that are difficult to weigh accurately such as those from very young mice and mice with developmental lens defects.

## Introduction

The ocular lens is a transparent tissue with a unique structure. The anterior surface of the lens comprises a single layer of cuboidal-shaped epithelial cells, while the bulk of the lens consists of elongated lens fiber cells that contain a high concentration of crystallin proteins. The entire lens mass is encapsulated in a basement membrane known as the capsule [1-3]. Murine lens growth occurs rapidly in the first three months of life, then levels off to a constant rate after about six months [2,4]. The epithelial layer contains three distinct zones of proliferative activity: 1) the central epithelium with the lowest mitotic activity, 2) the anterior equatorial zone (also known as the germinative zone) with the highest level of cell division, and 3) the transitional zone just posterior to the germinative zone, which lacks cell division and contains epithelial cells beginning to differentiate into the elongated fiber cells that will ultimately form the lens cortical ﬁbers [5-7]. In many animal models of lens developmental defects, the transitional zone has been found to be displaced posterior or anterior of the equator [8-10].

In our previous work, we studied the αA-crystallin R49C (αA-R49C) mouse model of lens development with epithelial layer abnormalities. We demonstrated that heterozygous or homozygous mutation of R49 to C in αA-crystallin causes lens opacities in mice [11,12]. These opacities appear at about 2 months of age in heterozygous mice, and at birth in homozygous mice. Qualitative analysis of lens histology, volume, and shape changes indicated a severely disrupted, though highly variable, lens phenotype in homozygous αA-R49C mice [13].

Due to the morphological lens defects in αA-R49C mice, the available methods for quantifying disruptions in lens structure for this model are fraught with inaccuracy. For example, biometric methods are commonly used to measure lens weight but do not assess the contribution of changes in lens structure to the overall defect [14]. Investigators have also developed study-specific methods for measuring the effects of culture and ﬁxation media on lens weight and dimensions [15], and the growth of the lens during aging in vitro [16-19]. However, these methods are ineffective for seriously disrupted, cataractous lens models in which the lens fragments during the removal process. We therefore developed a new method that can accurately quantify lens mass and shape from images of histological sections of small and/or grossly abnormal lenses from wild type, αA-R49C heterozygous, and αA-R49C homozygous mutant mice.

## Methods

### Animals and tissue

Mice were maintained at the Washington University Division of Comparative Medicine (St. Louis, MO) by trained veterinary staff. All protocols using animals were approved by the Animal Studies Committee, and followed institutional guidelines for use and care of animals in research comparable to guidelines established by the Institute for Laboratory Animal Research (Guide for the Care and Use of Laboratory Animals). Mice expressing the αA-R49C mutant protein were generated as previously described [11,12]. Heterozygous mice were interbred to produce wild type, heterozygous, and homozygous progeny. Homozygous mice were interbred to generate homozygous progeny. No lethality was associated with the mutation. Eyes were taken from mice sacrificed for a parallel study [13]. Eyes were freshly dissected, fixed, and used for histology. In some cases, fresh lenses were dissected, their surfaces dried briefly, and then weighed immediately. We analyzed a total of 76 lenses by histology. The age and genotype of these lenses are given in Table 1. Eyes were fixed immediately in 10% neutral-buffered formalin (Fisher Scientific, Pittsburgh, PA) and paraffin sections were processed for staining as previously described [13]. Bright ﬁeld images of sections were taken with an Olympus (Center Valley, PA) ﬂuorescence microscope ﬁtted with a digital camera (Spot Diagnostic Instruments, Sterling Heights, MI) as previously described [20]. Mice were examined by slit lamp biomicroscopy at various ages ranging from postnatal after eye opening to 9 months old. Slit lamp biomicroscopy was performed as previously described [12,21].

**Table 1:** Age and genotype of examined lenses.

** **	**Number of lenses**			
Age range	Wild type	Heterozygous	Homozygous	Total
0–2.5 weeks	10	10	21	41
2–6 months	6	13	3	22
9–12 months	1	10	2	13
Total	17	33	26	76

### Mass and volume calculation

**Figure 1:**
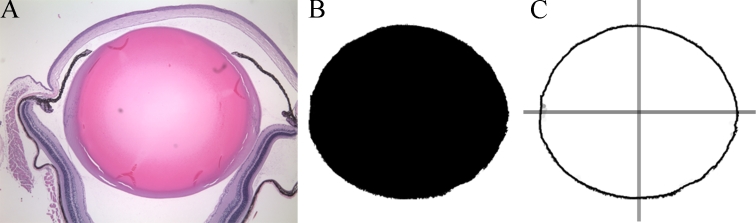
Illustration of the image analysis process. The histological section was photographed at high magnification (**A**). The pixels corresponding to the lens were then extracted (**B**) in Photoshop. Finally, the locations of the lens edge pixels were determined using an edge-finding algorithm in MATLAB (**C**). Overlaid lines in (**C**) indicate the locations of the optical axis (vertical line) and equator (horizontal line).

A mid-sagittal section with maximal equatorial diameter was aligned along the anterior-posterior axis. This technique allowed volume estimation to be performed repeatedly and reliably. To measure the lens volume, a digital photograph of a histological section through the optical axis was taken under high magniﬁcation (Figure 1A). The lens was then extracted from the image in PhotoShop CS3 using the Extract filter. An automated action was used to binarize the image by selecting the extracted area (i.e., the pixels corresponding to the lens), ﬁlling the area with black, and then filling the inverse selection with white (Figure 1B). The image was then saved in bitmap format.

MATLAB (The Mathworks, Inc.; Natick, MA) was used to objectively analyze the bitmap ﬁles. First, the image was split vertically along the optical axis into two sub-images (Figure 1C). The analysis applied the following steps separately to each sub-image and the results were averaged to improve accuracy. A Canny edge-ﬁnding ﬁlter was applied to the sub-image to determine which pixels corresponded to the edge of the lens. After the location of the edge pixels was established, their pixel coordinates were converted to actual dimensions using scale factors previously determined for the appropriate magnification. The actual edge coordinates were then used to compute the volume (*V*) using direct numeric integration with a multiple application trapezoidal rule [22]. This method assumed that the lens was rotationally symmetric about the optical axis. Thus, the volume was computed by integrating to find the cross-sectional area of the imaged section, then integrating over a 360° rotation. The numerical integration rule is as follows:

$$V=\frac{\pi }{2}\sum_{i=1}^{n}\left(x_{i+1}-x_{i}\right)(y^{2}{}_{i+1}-y^{2}{}_{i})$$

This is the discrete approximation of the volume integral. Here, *n* is the total number of edge pixels found in the sub-image and (*x*, *y*) are the coordinates of each edge pixel such that *x* is along the optical axis and *y* is in the direction perpendicular to the optical axis. This direct integration method allowed for inconsistent edge pixel spacing. Note that, while assuming axial symmetry for the lens is usually reasonable, potential errors arising in extreme cases are largely mitigated by performing the analysis on both halves of the globe and averaging the results.

This method gives an accurate measure of volume. Estimates of mass were obtained by multiplying the computed volume by a constant density of 1.0 g/ml solely to allow comparison to data from the literature for validation purposes. While changes in density within the lens or with age may occur, assuming a constant density is expected to give the most accurate results for comparison purposes. A second mouse model, the αA/αB-crystallin double knockout (DKO) model [23], was also analyzed to assess the applicability of our volume estimates to other models. Results from our method for wild type lenses were compared to the measurements by Rowe et al. [24] of dried lenses collected from mice of various ages.

### Shape characterization

The shape of the lenses was determined from histological cross sections, also using automated image analysis in MATLAB. The location of the edges was determined as for the volume calculation. The lens was divided into 360 equal angular sectors, taking the origin as the intersection of the optical axis with the equatorial plane. The distance from the origin to each edge pixel was computed and the mean distance value within each sector was recorded. Values from corresponding sectors of multiple lenses of similar age and genotype were averaged to give the final lens profile. This procedure was repeated for multiple ages of each genotype to analyze changes in lens shape during growth.

### Statistical analysis

Differences in lens mass and equatorial diameter were tested using a heteroscedastic *t*-test to compare groups of lenses with the same age but different genotypes. Statistical significance was taken as any p-value less than 0.05.

## Results

**Figure 2:**
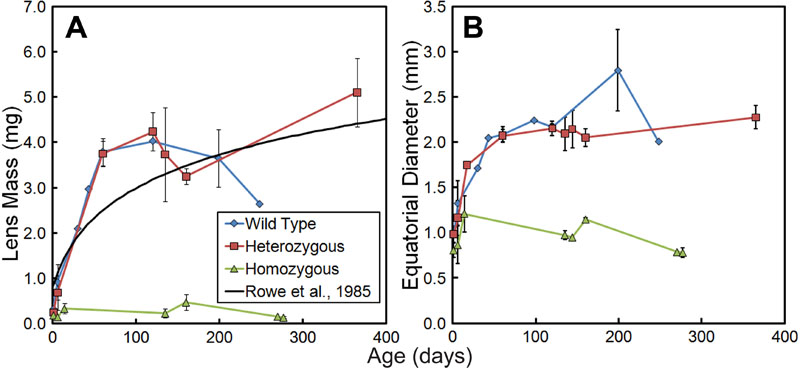
Measurements of lens mass and equatorial diameter. Mass (**A**) and equatorial diameter (**B**) of lenses from wild type and αA-R49C mutant mice determined using the proposed morphometric method. Error bars indicate standard deviation. Note that the calculated lens mass and equatorial diameter increased with age up to about 100 days in wild type and αA-R49C heterozygous but not in homozygous mutant mice.

Our morphometric method gave measurements comparable to the findings of Rowe et al. [24] for the dry weight age dependence of wild type lenses (Figure 2; R^2^=0.730). The mass and equatorial diameter of αA-R49C homozygous lenses were statistically lower than the wild type or αA-R49C heterozygous mutant lenses for all ages, whereas no significant difference between wild type and αA-R49C heterozygous lenses was found for any age. To evaluate the applicability of our method to other models, we analyzed lenses from αA/αB double knockout (DKO) mice [23], and observed significant differences in the calculated volume and mass between wild type and DKO lenses (p=0.011 and 0.002 for diameter and mass, respectively; data not shown). The mass of the αA-R49C homozygous lens did not differ from the mass of lenses from αA/αB DKO mice at birth, though the equatorial diameter was significantly larger in the αA-R49C homozygous lens. Wild type and heterozygous mouse lenses developed in a similar manner, and their growth rates were not statistically different. The wild type and heterozygous lens mass and diameter increased in a manner comparable to that reported [24] for dried lenses (R^2^=0.730). This indicated that the procedure used to fix the lenses dehydrated and artifactually reduced the size of the lenses. This finding was reinforced by the reported masses being lower than previously reported wet lens weights [25,26]. At all ages, both lens mass and equatorial diameters were significantly lower in the αA-R49C homozygous mice and increased very little with age compared to wild type or αA-R49C heterozygous mice. The agreement with the findings of Rowe et al. [24] indicates that the assumed density is not a significant source of error for most comparisons, whereas dehydrating fixation processes cause an underestimation of wet lens weight.

**Figure 3:**
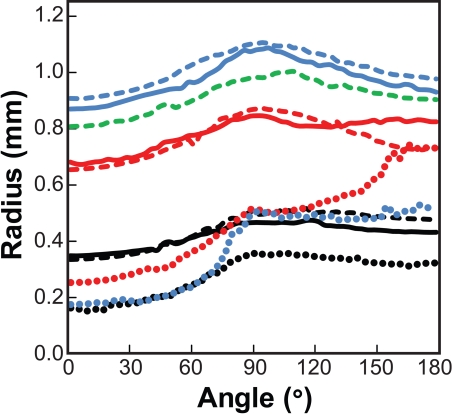
Measurement of lens shape. The radial dimension of the lens was measured from the intersection of the optical axis and the equatorial plane as a function of the angle measured from the anterior pole to the posterior pole for wild type, 6-day-old (black solid line); wild type, 3 months (red solid line); wild type, 4 months (blue solid line); heterozygous mutant αA-R49C, 6-day-old (black dashed line); heterozygous mutant αA-R49C, 2.5 weeks (red dashed line); heterozygous mutant αA-R49C, 3 months (blue dashed line); heterozygous mutant αA-R49C, 4 months (green dashed line); homozygous mutant αA-R49C, 6-day-old (black dotted line); homozygous mutant αA-R49C, 2 weeks (red dotted line); homozygous mutant αA-R49C, 4 months (blue dotted line). Note that wild type and αA-R49C heterozygous mutant lenses were nearly spherical with radial distances increasing slightly at the equator (angle=90°), whereas homozygous mutant lenses had a smaller anterior segment and an enlarged posterior segment.

Growth patterns were markedly dependent on both age and genotype (Figure 3). Wild type and heterozygous αA-R49C lenses displayed a nearly spherical shape, as indicated by a nearly constant radial distance with slight increases near the equator. Homozygous mutant lenses displayed a distinct shape with a smaller anterior segment and exaggerated posterior segment. The dimensions of the homozygous lens changed very little with age in contrast to the wild type and heterozygous lenses, but consistent with the mass measurements presented in Figure 2. The observed pattern of lens growth agrees with previous reports demonstrating disruption in the lens germinative zones caused by this mutation [13].

**Figure 4:**
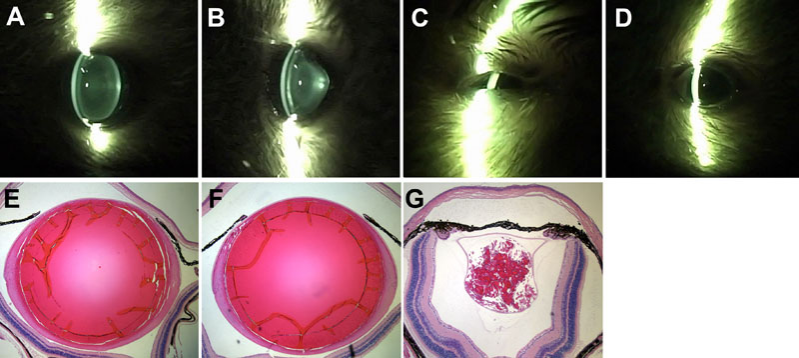
Qualitative phenotype comparison of wild type and αA-R49C mutant mouse eyes. Nine-month-old mice were examined using slit-lamp microscopy (above) and histology (below).  The images shown are from wild type (**A** and **E**), αA-R49C heterozygous mutant (**B** and **F**), and αA-R49C homozygous mutant (**C**, **D**, and **G**) mutant mice.  Mid-sagittal sections were stained with hematoxylin and eosin. Note the ocular defects in homozygous mutant mice (**C**, **D**, and **G**).

To further evaluate the effect of age on lens defects in αA-R49C homozygous mice, eyes were examined by histology (Figure 4). Lens fiber mass was significantly lower in αA-R49C homozygous eyes at all ages. We used a method similar to that shown in Figure 1 to map the severely disrupted lens in Figure 4G, with the capsule as the boundary of the lens. The αA-R49C homozygous mice developed defects in the iris that extended across the anterior face of the lens (Figure 4C,G). These images represent the most severe phenotypes in the homozygous mouse. The image in Figure 4C was taken with the lid of the right eye of a homozygous animal fully open. The lids appear to be partially closed because the eye is so much smaller than a normal wild type eye. Despite the reduced eye size, the histology clearly indicates a lens is present (Figure 4G). The absence of visible scattering in the lens in Figure 4C is caused by closure of the pupil, with the lens becoming obscured. In other homozygous animals, the lens was completely absent. The image in Figure 4D shows the remnant of the lens in the nasal quadrant of the left eye.

## Discussion

Patients heterozygous for the αA-crystallin R49C mutation develop lens opacities during infancy or at birth [27]. We have analyzed the effect of the mutation on lens opacities in gene knock-in mice [11,12]. The present study extends our previous work by describing the detailed histological characteristics of the αA-crystallin R49C mutation-associated opacities. In addition, the present study details new quantitative morphometric methods for assessing changes in shape, lens mass, and volume for small or grossly abnormal eyes.

We have previously shown that knock-in mice homozygous for the R49C mutant allele of αA-crystallin have much smaller lenses than their heterozygous mutant and wild type counterparts. The αA-R49C mutation causes opacification as a result of protein insolubility [11,12], and in homozygous mutant lenses the accumulation of insoluble protein appears to cause extensive cell death. Although early fiber cell differentiation is not significantly impacted, the deeper cortical fibers exhibit a dramatically disrupted morphology [12,13].

The average mass of a fresh wild type mouse lens calculated using a biometric method was reported to be 1.6 mg at 6 days of age [28]. Using our new morphometric method presented in this report, we calculated a mass of 1.1–1.2 mg for 6-day-old fixed wild type lenses. This difference between these measurement is consistent with the difference in mass between fresh and fixed lenses [15]. Investigators have measured lens shape with other biometric methods using isolated lenses [19]. Fixation causes about a 25% decrease in the wet weight of human lenses due to dehydration. Loss of water from the lens cortex and storage of fresh lenses causes a failure of volume regulating mechanisms, which stabilize within 3 weeks [16]. Consistent with these results, our measurements of histological sections generated values that were approximately 25% lower than fresh weights [14,29].

Our new morphometric method provides reproducible and quantitative measurements of lens volume that are useful for comparing the growth of ocular tissues from very young mice, and mice with disrupted and small lenses that are difficult to weigh accurately. In this method, the volume measurement accurately represents the total growth of the lens (Figure 2), whereas the specific geometry of the lens (Figure 3) gives a more accurate measurement of how the growth of the mutant lens diverges from that of the wild-type. To map the lens in Figure 4G, the capsule was used as the boundary of the lens. The choice to use the capsule as the boundary of the lens necessarily impacts the reported mass and shape values. This choice is based on the entire set of observations of the lens. If the preponderance of observations indicates that the in vivo lens fills the capsular bag without voids, and that voids appear due to processing, then the capsule may be considered to represent the true boundary of the lens. If instead the voids arise due to developmental phenotype observed via slit-lamp or other methods, then the boundary of the lens mass should be chosen for these measurements.

Quantitative biometric methods like ours provide more accurate determination of the age of phenotype onset. They also permit a more comprehensive view of the macroscopic changes occurring in the lens. In this study, we describe new morphometric methods for analysis of fixed eyes that may be used to quantitatively examine variation in lens shape and location of various cell types within the lens. These tools allow objective, quantitative comparison of these factors between genotypes, and facilitate detection of subtle phenotypic differences to yield greater physiologic insight.
